# A description of HIV prevalence trends in Nigeria from 2001 to 2010: what is the progress, where is the problem?

**DOI:** 10.11694/pamj.supp.2014.18.1.4608

**Published:** 2014-07-21

**Authors:** Adebobola Bashorun, Patrick Nguku, Issa Kawu, Evelyn Ngige, Adeniyi Ogundiran, Kabir Sabitu, Abdulsalam Nasidi, Peter Nsubuga

**Affiliations:** 1 Nigeria Field Epidemiology and Laboratory Training Program (N-FELTP), Abuja, Nigeria; 2 HIV/AIDS Division, Department of Public Health, Federal Ministry of Health, Nigeria; 3 World Health Organization, Nigeria Office; 4 Department of Community Medicine, Ahmadu Bello University Zaria, Nigeria; 5 Nigeria Center for Disease Control and Prevention (NCDC), Abuja, Nigeria; 6 Global Public Health Solutions, Decatur, GA, USA

**Keywords:** Sentinel, HIV, Pregnant, Nigeria, antenatal, clinic, prevalence, estimates, projection, testing

## Abstract

**Introduction:**

Nigeria's population of 160 million and estimated HIV prevalence of 3.34% (2011) makes Nigeria the second highest HIV burden worldwide, with 3.2 million people living with HIV (PLHIV). In 2010, US government spent about US$456.5 million on the Nigerian epidemic. Antenatal clinic (ANC) HIV sero-prevalence sentinel survey has been conducted biennially in Nigeria since 1991 to track the epidemic. This study looked at the trends of HIV in Nigeria over the last decade to identify progress and needs.

**Methods:**

We conducted description of HIV sero-prevalence sentinel cross-sectional surveys conducted among pregnant women attending ANC from 2001 to 2010, which uses consecutive sampling and unlinked-anonymous HIV testing (UAT) in160 sentinel facilities. 36,000 blood samples were collected and tested. We used Epi-Info to determine national and state HIV prevalence and trends. The Estimation and Projection Package with Spectrum were used to estimate/project the burden of infection.

**Results:**

National ANC HIV prevalence rose from 1.8% (1991) to 5.8% (2001) and dropped to 4.1% (2010). Since 2001, states in the center, and south of Nigeria had higher prevalence than the rest, with Benue and Cross Rivers notable. Benue was highest in 2001 (14%), 2005 (10%), and 2010 (12.7%). Overall, eight states (21.6%) showed increased HIV prevalence while six states (16.2%) had an absolute reduction of at least 2% from 2001 to 2010. In 2010, Nigeria was estimated to have 3.19 million PLHIV, with the general population prevalence projected to drop from 3.34% in 2011 to 3.27% in 2012.

**Conclusion:**

Examining a decade of HIV ANC surveillance in Nigeria revealed important differences in the epidemic in states that need to be examined further to reveal key drivers that can be used to target future interventions.

## Introduction

According to the 2012 Joint United Nations Program on HIV/AIDS (UNAIDS) report on the state of the HIV epidemic globally, Nigeria is among the 12 countries that experienced a stable (i.e., less than 25% change up or down) rate of HIV infection within the decade 2001-2010 [1]. However, several neighboring countries to Nigeria have experienced drops of their incidence rates from 2001 to 2010 of at least 25% and others e.g., Central African Republic and Ghana have greater than 50% drop in incidence rates [1]. Since 1991, Nigeria has used the antenatal clinic (ANC) HIV sero-prevalence sentinel surveillance (HSSS) to track the HIV epidemic. Pregnant women constitute the most practical group for this survey as they are sexually active, easily defined and accessible, and are receiving care, which requires routine blood drawn for syphilis testing. Pregnant women are also generally representative of the sexually active population. It is estimated that about 54.5%-60% of pregnant women had at least one ANC visit even though there are extreme variations in the different states and among social classes in Nigeria [2, 3]. The HSSS conducted in 2010 showed variations in HIV prevalence in Nigeria [4]. The combination of Nigeria's population size, projected at >160 million by the World Bank in 2011 [5] and estimated HIV prevalence of 3.34% (ages 15-49 years) for the same year [4], is the second highest burden of HIV/AIDS worldwide, with an estimated 3.2 million people living with HIV in 2011 and an estimated 2.4 million children orphaned by HIV/AIDS [4]. Nigeria also has one of the highest tuberculosis burdens in the world (i.e. >300 cases/100,000 population) and the largest in Africa [6]. Nigeria has a generalized HIV epidemic but prevalence varies widely across states and locations [4, 7]. The key drivers of the Nigeria HIV epidemic are heterosexual transmission and mother-to-child transmission. Despite the rapid expansion of HIV/AIDS services across the country, coverage of essential prevention and treatment interventions remains low, and the level of unmet demand is high [6].

The period from 1990 to 2007 experienced a large rise in global health funding from all global health donors both public and private [8]. Nigeria has been shown to be second only to India in the amount of global health funding [9] and continued to receive significant donor support to address its HIV/AIDS epidemic and it received almost US $ 442 and US $ 457million for HIV/AIDS programs from the US government in 2009 and 2010 respectively [10]. The recent global financial recession could very well lead to a reduction in the donor funds available for health [11], hence a need to have a careful look at the results being generated with these funds. Nigeria like other developing countries is in the midst of an epidemiologic transition [12, 13] and is a diverse country with many tribes, regions/states, and varying HIV prevalence. Identifying and exploring differences in states’ HIV/AIDS prevalence and programming could lead to a determination of key drivers for success in those states that have reduced HIV prevalence levels. Conversely, determining the factors that have contributed to a high or increasing level of HIV prevalence could provide lessons on what to avoid. We looked at the trends of HIV in Nigeria over the decade of 2001 to 2010 as described in the 2010 ANC sentinel surveillance report [4] and conducted more analysis to identify where progress has been made and where efforts need to be redoubled in order to make recommendations to policy makers.

## Methods

We conducted a description of ANC HSSS from 2001-2010 in Nigeria. A cross-sectional study design is being used to conduct the HSSS among pregnant women attending ANC for the first time for that pregnancy [14]. Nigeria uses consecutive sampling and the unlinked anonymous testing (UAT) method of sample collection and HIV testing based on World Health Organization (WHO) recommendations [14]. For the surveys from 2001-2010, each year's data were collected over a period of 12 weeks from 160 sentinel sites in the 36 states in Nigeria and the Federal Capital Territory (FCT). The previous years’ surveys have used the same methodology, sampling pregnant women who were attending antenatal clinics for the first time for a confirmed pregnancy, aged 15-49 years old. That for 2010 was expanded disregarding age. Every round of HSSS recruited approximately 36,000 pregnant women, and in 2010, a total of 36,427 blood samples were collected and analyzed. The selection of survey sites was based on the following criteria: participation in previous surveys, availability of staff and facilities required for drawing blood from antenatal clinic attendees on their first visit of the current pregnancy, provision of services to a relatively large number of pregnant women per week to meet the minimum sample size in 12 weeks, and availability of qualified personnel and willingness of on-site staff to cooperate. For each selected site, all relevant personnel were identified and trained. With a minimum of two urban and two rural sites per state and FCT, a total of 160 sites comprising 86 urban and 74 rural were used for the survey. Based on WHO recommendations [14], which took into consideration an estimate of HIV prevalence in the population to be surveyed, the precision or relative error considered acceptable is 0.05 and a desired level of confidence is 95%, a minimum sample size of 300 was deemed adequate per selected site, using the formula below:

N = Z2pq/d2. Where: N = Minimum sample size required Z= Standard normal deviate, usually set at 1.96 which correspond to a 95%confidence interval P = Proportion in the target population estimated to have a particular characteristic which in this case is 5% (which is the estimated HIV infection prevalence) q = 1-p d= degree of accuracy set at 0.025 Substituting n = 1.962 X 0.05 X 0.95/0.0252= 291.

The sample size was increased to 300 to make up for any data or specimen loss during the survey. The two rural sites in each state generated a minimum combined sample size of 300 (each of the rural site has 150) such that the rural samples form a rural cluster with a total sample size large enough to be analyzed by state. This was used to estimate the rural prevalence in each state. In a site, all eligible women who attended antenatal clinics for booking during the survey period were consecutively enrolled till sample size is met. Definitive steps were taken to ensure that specimens were properly de-linked. After syphilis testing at the site, all identifiers of the survey specimens were removed and destroyed before transportation to the state central laboratory for survey HIV testing. This survey protocol was ethically approved by both the National Health Research Ethics Committee of Nigeria as well as the Institutional Review process of the US Centers for Disease Control and Prevention (CDC). Epi Info software was used to derive estimates and distribution of the mean figures for HIV prevalence amongst ANC attendees in each state and to calculate the median figure for the national HIV prevalence. The methods, tools and assumptions used to estimate the burden of HIV/ AIDS in Nigeria are based on the recommendations made by the UNAIDS Reference Group on estimates, modeling and projections [14]. The Estimation and Projection Package (EPP 2009) was used to estimate and project adult HIV prevalence and the burden of infection in the country from the surveillance data obtained from ANC clients [15]. The 2009 version of EPP used HIV treatment data, base population, sex ratio and urban rural infection ratio to improve the estimation of incidence from the prevalence over time. EPP is used to fit a simple epidemic model to data from urban and rural sites. The resulting national estimated adult HIV prevalence was then transferred to a demographic package, Spectrum 2009, using the AIDS Impact Model for demographic projections to calculate the number of people infected and other parameters, such as AIDS cases, AIDS deaths and AIDS orphans. The estimates were based on the assumption that Nigeria's population in 2006 was 140 million [16], that a significant percentage of persons (i.e., 34%) who require antiretroviral therapy were receiving such treatment [17], and that some efforts were being made to provide Prevention of Mother-to-Child Transmission services to the population who live in the rural areas. Comparisons and trends assessment/analysis were further conducted on the data for the study periods 2001-2010, due to the very similar methodologies used to conduct the ANC sero sentinel survey in the last five rounds. An absolute reduction in prevalence (additive) and not a relative (multiplicative) reduction were considered during the analysis.

## Results

**Figure 1:**
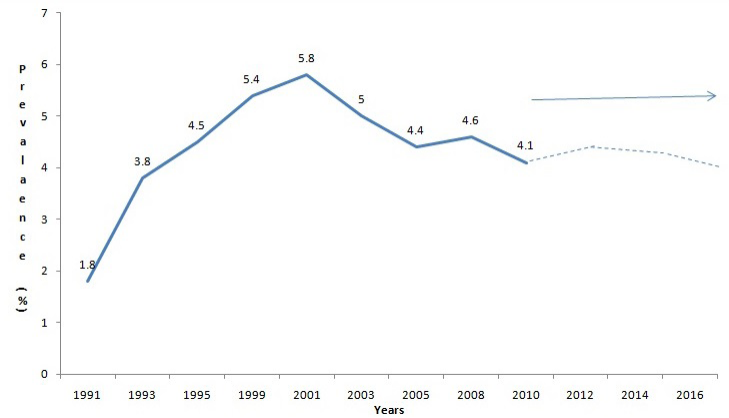
National HIV Prevalence Trend from 1991 to 2010, Nigeria. Source: Nigeria ANC Surveillance Report 2010, Federal Ministry of Health, Nigeria

**Figure 2:**
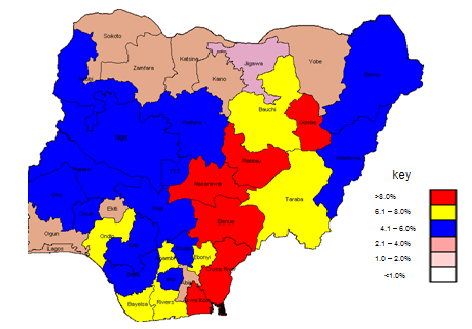
Prevalence of HIV by State Nigeria, 2001, Source: Nigeria ANC Surveillance Report 2001, Federal Ministry of Health, Nigeria

Figure 1 shows the national HIV prevalence from 1991-2010 using the median values of prevalences in the 36 states and the FCT. Beginning with a less than 2% estimated HIV prevalence in 1991, the national estimated HIV prevalence rose steadily by year to a high of 5.8% in 2001. The estimated HIV prevalence dropped from 2005 to 2008 and then plateaued to between 4.4% and 4.1% by 2010. In 2001 the HIV epidemic in Nigeria was present in all the states. The estimated prevalence in 2001 ranged from 1.8% -13.5% with a median prevalence of 5.2%. The highest rates above 8% were in Gombe, Plateau, Nasarawa, Benue, Cross Rivers, Akwa-Ibom and FCT with Benue having highest prevalence at 13.5%. All the northern-most states and the south-eastern states had lower rates below 5.0%, with a northern-most state, Jigawa state with the lowest at 1.8% (Figure 2).

**Figure 3:**
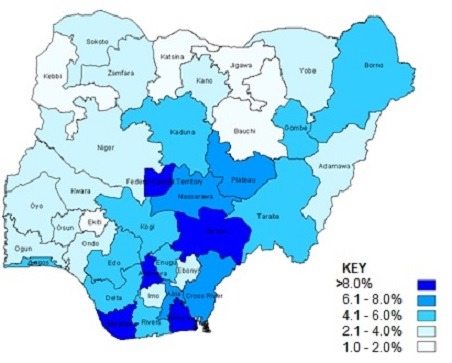
Prevalence of HIV by State in 2010, Source: Nigeria ANC Surveillance Report 2010, Federal Ministry of Health, Nigeria

In 2005 the estimated HIV prevalence ranged from 1.6% in Ekiti to 10% in Benue with a median of 4.0%. In 2010, the HIV epidemic in Nigeria was still generalized across the country. The prevalence ranged from 1.0% -12.7% with a median prevalence of 4.1%. States in the south and center of the country had the highest rates and states in the north of the country had the lowest rates. Bayelsa, Akwa-Ibom, Anambra, Benue and FCT had prevalence values above 8.0% (Figure 3), with Benue highest with prevalence of 12.7%. Three states Kebbi, Jigawa and Ekiti had below 2.0% with lowest prevalence of 1.0% while Jigawa had 1.5%. Table 1(a) and Table 1(b) show comparisons of HIV prevalence by state by year. Benue, consistently had the highest rate among all states from 2001 through 2010, while Ekiti and Jigawa were consistently below 2.0% while Abia's prevalence that was below 5.0% in previous years rose to 7.3% in 2010. Comparing 2001 to 2010, eight states (21.6%) out of 36 states and FCT showed an increased estimated HIV prevalence from 2001 to 2010, the rest of the states had a reduced estimated prevalence or were stable between the 2001 and 2010 estimate. Conversely six states (16.2%) had a reduction of the estimated prevalence of at least 2% from 2001 to 2010. Three states (8.1%) (Bauchi, Gombe, and Ondo) had a reduction of the estimated HIV prevalence of 4% or higher between 2001 and 2010.

Comparing 2008 to 2010, in 19 (51.3%) out of 36 states and FCT, the estimated HIV prevalence in 2010 was higher than the estimated HIV prevalence in 2008 (i.e., short term reversal of any gains made in 2008 thereby a short term upward trend) (Table 1(a) and Table 1(b)).

**Table 1(a):** Prevalence of HIV by State from 2001 to 2010, with change in prevalence from 2001 to 2010, and from 2008 to 2010

State	2001	2003	2005	2008	2010	Change from 2001 to 2010*	Change from 2008 to 2010
Abia	3.3	3.7	4	5	7.3	4	2.3
Anambra	6.5	3.8	4.2	5.6	8.7	2.2	3.1
Bayelsa	7.2	4	3.8	7.2	9.1	1.9	1.9
Lagos	3.5	4.7	3.3	5.1	5.1	1.6	0
Borno	4.5	3.2	3.6	2	5.6	1.1	3.6
Sokoto	2.8	4.5	3.2	6	3.3	0.5	-2.7
AkwaIbom	10.7	7.2	8	9.7	10.9	0.2	1.2
Kogi	5.7	5.7	5.5	5.1	5.8	0.1	0.7
Enugu	5.2	4.9	6.5	5.8	5.1	-0.1	-0.7
Jigawa	1.8	2	1.8	1.6	1.5	-0.3	-0.1
Edo	5.7	4.3	4.6	5.2	5.3	-0.4	0.1
Kano	3.8	4.1	3.4	2.2	3.4	-0.4	1.2
Ogun	3.5	1.5	3.6	1.7	3.1	-0.4	1.4
Taraba	6.2	6	6.1	5.2	5.8	-0.4	0.6
Kaduna	5.6	6	5.6	7	5.1	-0.5	-1.9
Niger	4.5	7	5.3	6.2	4	-0.5	-2.2
Nasarawa	8.1	6.5	6.7	10	7.5	-0.6	-2.5
Adamawa	4.5	7.6	4.2	6.8	3.8	-0.7	-3
Benue	13.5	9.3	10	10.6	12.7	-0.8	2.1
Plateau	8.5	6.3	4.9	2.6	7.7	-0.8	5.1
Cross River	8	12	6.1	8	7.1	-0.9	-0.9

Table sorted by absolute change in Prevalence from 2001-2010

**Table 1(b):** Prevalence of HIV by State from 2001 to 2010, with change in prevalence from 2001 to 2010, and from 2008 to 2010

State	2001	2003	2005	2008	2010	Change from 2001 to 2010 *	Change from 2008 to 2010
Oyo	4.2	3.9	1.8	2.2	3	-1.2	0.8
Imo	4.3	3.1	3.9	4.6	3	-1.3	-1.6
Yobe	3.5	3.8	3.7	2.7	2.1	-1.4	-0.6
Zamfara	3.5	3.3	3	2.1	2.1	-1.4	0
Katsina	3.5	2.8	2.7	2.6	2	-1.5	-0.6
Osun	4.3	1.2	2	1.2	2.7	-1.6	1.5
FCT	10.2	8.4	6.3	9.9	8.6	-1.6	-1.3
Delta	5.8	5	3.7	3.7	4.1	-1.7	0.4
Rivers	7.7	6.6	5.4	7.3	6	-1.7	-1.3
Ekiti	3.2	2	1.6	1	1.4	-1.8	0.4
Kwara	4.3	2.7	2.8	1.8	2.2	-2.1	0.4
Ebonyi	6.2	4.5	4.5	2.8	3.3	-2.9	0.5
Kebbi	4	2.5	4	2.9	1	-3	-1.9
Gombe	8.2	6.8	4.9	4	4.2	-4	0.2
Ondo	6.7	2.3	3.2	2.4	2.3	-4.4	-0.1
Bauchi	6.8	4.8	3.4	3.1	2	-4.8	-1.1
**National**	5.8	5	4.4	4.6	4.1	-1.7	-0.5

Table 1b is a continuation of Table 1a and is also sorted by absolute change in Prevalence from 2001-2010

Three states (Bauchi, Kebbi, and Ondo) (8.1% of 36 states and FCT) had both a reduced estimated HIV prevalence of 3% or higher between 2001 and 2010 and a reduction of their estimated HIV prevalence in 2010 when compared to 2008 (i.e., long term maintenance of gains therefore a long term downward trend). Results from EPP/spectrum analysis in 2010, showed that Nigeria would have a total of 3.19 million HIV infected people in 2012, with the majority adult females. The estimated national HIV prevalence among the 15-49 year old age group was projected to have dropped from 3.34% in 2011 to 3.27% in 2012 (Table 2). HIV treatment was projected to be required for 1.58 million people in 2012 of which 217,000 were children <15 years old. A total of 285,270 new HIV infections were expected in 2012 of which 157,510 were new infections in children. There was a projected reduction in annual deaths in 2012 from 2011. The total number of AIDS orphans was projected to rise to 2.527 million in 2012 from 2.229 million in 2010.

**Table 2:** HIV estimates and projections

HIV Estimates and Projections	2010	2011	2012
**HIV population (all)**			
Total	3,140,000	3,150,000	3,190,000
Males	1,320,000	1,320,000	1,340,000
Females	1,820,000	1,830,000	1,850,000
Adults(≥15yrs)	2,810,000	2,820,000	2,850,000
Male	1,150,000	1,150,000	1,170,000
Female	1,660,000	1,670,000	1,680,000
Total Children(<15yrs)	321,580	331,150	338,010
Male	163,600	168,480	171,970
Female	157,980	162,670	166,040
Prevalence(15-49)	3.42	3.34	3.27
**Cumulative AIDS Deaths**			
Total	2,100,000	2,340,000	2,560,000
Males	970,000	1,080,000	1,180,000
Females	1,130,000	1,260,000	1,380,000
**Annual AIDS Deaths (Yearly)**			
Total	215,130	233,170	218,160
Males	96,740	104,900	97,680
Females	118,390	128,270	120,480
**ART Programme**			
Total requiring ART(Adults)	1,300,000	1,340,000	1,360,000
Total requiring ART(<15yrs)	212,720	215,780	217,750
All requiring ART	1,512,720	1,555,780	1,577,750
**New HIV Infections**			
Total New Infections	281,180	284,220	285,270
Adult New Infections	126,260	127,430	127,760
Childhood New I nfections	154,920	156,790	157,510
**Total number of children (<15yrs) orphaned due to HIV/AIDS**			
Total AIDS Orphans	2,229,883	2,419,984	2,527,102
Maternal AIDS Orphans	1,810,703	1,942,000	1,998,751
Paternal AIDS Orphans	1,401,481	1,521,736	1,592,226
Dual Orphans	1,199,833	1,273,590	1,296,765

Source: Nigeria ANC Surveillance Report 2010, Federal Ministry of Health, Nigeria. Estimates derived through use of EPP and Spectrum software.

## Discussion

The HIV/AIDS epidemic is one of the major public health challenges faced by Nigeria. The HIV prevalence figures derived in these surveys conducted over the years are from pregnant women attending antenatal clinics, with a stabilizing prevalence of about 4% in the last three sentinel surveys. This has been used over the years as proxy for the HIV prevalence in the general populace. In our examination of the HIV prevalence in Nigeria for the decade 2001 to 2010 using the 2010 ANC surveillance report and data, we found the following three key issues. First, the overall HIV prevalence in Nigeria plateaued between 4% and 5% in the second half of the decade. Second, there were important differences in the state to state comparisons, with some states maintaining a long term reduction of their HIV prevalence between 2001 and 2008 by 2010, while others showed a reversal of any gains they had made between 2001 and 2008 by 2010. Third, the number of HIV-infected people who will need care and treatment and by inference the number of Nigerians who will need prevention from being infected by HIV is expected to continue to rise. The result of the 2010 HIV sentinel surveillance among pregnant women attending antenatal clinics showed that the epidemic affected all the geopolitical zones, states, urban and rural locations in the country with very wide variations. With a median prevalence of 4.1%, it is estimated that over 3.1 million Nigerians are currently infected with the virus and about 1,512,720 would require treatment. Based on this, there is a need for all the stakeholders in the country to maintain and scale up the current momentum of interventions. Even though there is a decrease in the national median value for prevalence from 2008 to 2010, findings that 19 out of the 37 states (including FCT) have a higher mean value for prevalence in 2010 than they had in 2008 should be concerning, while 16 have a reduction and only three maintained the 2008 value in 2010. This implies that there may be some underlying changes in HIV programming that in effect are delivering sub-optimal results in these states with increasing value. A higher prevalence may signify fewer people dying from HIV which is good (survival bias) or more likely that more people are being infected which is a bad hypothesis. In any case the cause of these figures needs to be investigated urgently. An incidence study will also be relevant at this point. Qualitative studies should be done to compare states that are experiencing consistent reductions like Bauchi, Kebbi, and Ondo with other similar states that are experiencing increases to discover what lesson can be learned. This kind of in depth ethnographic operational research will help to target interventions to where they may be most useful using in country exemplars in a peer to peer relationship. Over several years of HIV ANC surveillance, some states have apparently traditional “hot spots” (i.e., areas with high HIV prevalence). Anecdotal information supports some hypothesized relationship of local risk factors associated with “hot spots” in those states. Similarly, there are relatively regular “cold spots” (i.e., areas with low prevalence) across the country. This observation of persistent “hot spots” and “cold spots”′ seems to defy explanation in terms of the impact of interventions but can be studied and exploited for better programming. Some countries like Malawi have experienced plateauing epidemics and have pointed out that this could be driven by persisting risky behaviors, whether this is true or not in Nigeria needs to be investigated [18].

Many countries are considering offering early lifetime HIV treatment for all HIV infected patients or for all HIV infected pregnant women as a form of prevention-the logic being that treating all HIV infected people will reduce partner infections and vertical transmission. Based on the work conducted in several countries by Cohen et al in 2007 to 2010 [19], early treatment as prevention is highly efficacious. Early treatment as prevention also offers simplified programming which eliminates the need for costly or inaccessible CD4 tests and possibly confusing treatment algorithms. However treatment as prevention is predicated on a high level of access to HIV testing and it is also unclear how much it would cost or how acceptable it would be in Nigeria, in any case treatment as prevention is an option that needs to be urgently studied to determine its feasibility and effectiveness in Nigeria. In order to treat the projected number of HIV-infected people in Nigeria in 2012, and future years, a national scale up is needed. In order for this to happen, all HIV testing including those done in UAT surveys have to be linked so that the identified respondents and pregnant women can access antiretroviral drugs. Also for ethical reasons we know that it is unethical to deny those that will benefit from a treatment access to the treatment since it is available. This will further scale up treatment. Another important issue is the need to develop a strengthened sustainable health system in Nigeria, with all the six WHO identified building blocks (i.e., health service delivery, human resources for health, medical products, vaccines and technologies, information, leadership and governance, and financing) receiving adequate emphasis [20]. Sustaining current and previous investments in HIV programing hinges on leadership and national ownership as well as national (i.e., domestically sourced) financing from the national coffers. Integration of health systems strengthening efforts including health data sources is critical for both HIV and other non HIV interventions. It is recommended that risk factors and programming related factors responsible for differences in state to state HIV/AIDS prevalence should be identified and explored to determine key drivers for success in those states that have reduced HIV prevalence levels and key drivers for those that have a high or increasing level of HIV prevalence so as to guide appropriate interventions.

One of the limitations of ANC sentinel surveillance is the fact that women attending public health facilities may not be representative of women in the general population since the latter includes those who are using some form of contraception, as well as those who are infertile, and those who refuse to come for some reasons including medical such as already known HIV status; and also because of their acceptance of traditional beliefs and patronage of traditional birth attendants (TBA). This surveillance is done in public institutions only, so may not be representative for those that patronize private institutions. Moreover, pregnant women who choose to attend public health facilities may have Socio-economic characteristics different from those that attend private institutions; and a substantial proportion of pregnant women, for various reasons, may not attend antenatal clinics. It is also known that men and women have different HIV-related risk behaviors and therefore may have different rates of infection. The HSSS excludes men. Prior to the 2010 round of survey, it focused on ages 15-49 years only, but even the expansion in age boundary for 2010 had no effect on prevalence. Sentinel sites were purposefully selected on the basis of specific criteria and therefore may not be representative of all the health facilities. Among the selected facilities, there may be policies and practices that may influence the pattern of attendance at ANC. However, studies in many countries have shown that HIV prevalence from pregnant women compares favorably with data from the general population though with some caution [21, 22]. The survey does not have comprehensive behavioural study component in its design and methodologies. The variables on behaviors and attitudes are very few (about eight variables on socio-demographics). There have also been concerns on the expediency of conducting sentinel surveys using the unlinked anonymous testing (UAT) approach. The argument is that with the availability of HIV programing data (e.g.treatment and prevention of mother to child transmission (PMTCT) services, PMTCT data can be used to replace UAT. In countries like Botswana and Thailand where ANC and PMTCT coverage are high and routinely reported PMTCT data are complete and accurate [23–25], PMTCT can be used to track HIV prevalence in the general population instead of using UAT which is based on ANC data.

In Nigeria, despite the efforts aimed at training staff on PMTCT data collection, analysis and reporting, it is observed that routinely reported PMTCT data are still of poor quality. This may point to the need for general health system strengthening to improve all the data streams within health. In 2010 and as 2012, the number of PMTCT sites was approximately 700 while the proportion of pregnant women covered in PMTCT was between 14-21% [17, 26]. Effort to assess the utility of PMTCT service data as a substitute for the data from ANC surveillance has commenced in Nigeria and the effort is ongoing. The prevalence ratios between those who accept HIV testing and those who refuse are not known. In view of these potential sources of bias in comparing ANC HIV prevalence data with routinely reported PMTCT prevalence data, it appears that anonymous testing should continue to serve as a reliable method of monitoring HIV prevalence in the general population. Though the general population based surveys provide better prevalence estimates (including for men) they are quite expensive and could remove much needed resources from HIV programming.

## Conclusion

Examining a decade of HIV ANC HSSS in Nigeria revealed important differences in the country-wide epidemic by state and location. The reversal of downward trends and the constantly high prevalence in some states is worrisome. This need to be examined further to reveal key drivers of the epidemic that can be targeted for interventions in the future. Implementing new interventions such as early HIV treatment as prevention may be one strategy that may lead to a downward national HIV prevalence trend from the current level. The success in implementing new interventions will hinge on a strengthened and ably led health system. Furthermore, this should be financed adequately from national coffers while leveraging on international support and using evidence based interventions to achieve desirable targets. The potential threat of inappropriate action may lead to explosion of HIV incidence to the traditionally low prevalence states which will raise the national prevalence significantly. Further studies to determine risk factors and programming issues responsible for national prevalence changes, variations in states, and locations should be conducted so as to determine the key drivers for success, and the drivers of epidemics that may be ameliorated so as to guide the design of appropriate interventions for the country.

## References

[1] UNAIDS (2012). UNAIDS report on the global AIDS epidemic 2012.

[2] National Population Commission (2008). Nigerian Demographic and Health survey.

[3] The World Bank (2008). WB_Nigeria report.

[4] Federal Ministry of Health (FMOH) (2010). Technical report on National HIV Sero-prevalence sentinel Survey Among Pregnant Women Attending Antenatal Clinics in Nigeria.

[5] The World Bank (2013). DataBank, World Development Indicators (Internet). http://databank.worldbank.org/data/views/reports/tableview.aspx.

[6] WHO/UNAIDS (2007). Towards Universal Access ACCESS Scaling up priority HIV/AIDS interventions in the health sector Progress Report.

[7] Federal Ministry of Health (FMOH) (2010). 2010 Nigeria Integrated Biological and Behavioural Surveillance Survey.

[8] Ravishankar N, Gubbins P, Cooley RJ (2009). Financing of global health:tracking development assistance for health from 1990 to 2007. Lancet..

[9] Christopher Murray JL, Brent Anderson, Roy Burstein, Katherine Leach-Kemon, Matthew Schneider, Annette Tardif, Raymond Zhang (2011). Development assistance for health: trends and prospects. The Lancet.

[10] PEPFAR (2012). Nigeria Operational Plan Report FY 2010 (Internet). http://www.pepfar.gov/documents/organization/145730.pdf.

[11] Joses Kirigia M, Benjamin Nganda M, Chris Mwikisa N, Bernardino Cardoso (2011). Effects of global financial crisis on funding for health development in nineteen countries of the WHO African Region. BMC International Health and Human Rights (Internet).

[12] World Health Organization, Organization WH (2009). Global health risks: mortality and burden of disease attributable to selected major risks.

[13] Steyn K, Damasceno A, Dean Jamison T, Richard Feachem G, Malegapuru Makgoba W (2006). Lifestyle and Related Risk Factors for Chronic Diseases. Disease and Mortality in Sub-Saharan Africa.

[14] UNAIDS/WHO Working Group on Global HIV/AIDS and STI Surveillance (2003). Guidelines for Conducting HIV Sentinel Serosurveys among Pregnant Women and Other Groups.

[15] Stover J, Johnson P, Zaba B, Zwahlen M, Dabis F, REE (2008). The Spectrum projection package: improvements in estimating mortality, ART needs, PMTCT impact and uncertainty bounds. BMJ, Sex Transm Infect.

[16] National Population Commission (2006). Official Census Results:Nigeria Has 140 Million People (Internet). http://www.population.gov.ng.

[17] UNAIDS/NACA (2010). United Nations General Assembly Special Session (UNGASS) Country Progress report.

[18] Bello GA, Chipeta J, Aberle-Grasse J (2006). Assessment of trends in biological and behavioural surveillance data: is there any evidence of declining HIV prevalence or incidence in Malawi?. Sex Transm Infect..

[19] Cohen MS, Chen YQ, McCauley M (2011). Prevention of HIV-1 Infection with Early Antiretroviral Therapy. N Engl J Med.

[20] World Health Organization (2010). Monitoring the building blocks of health systems: a handbook of indicators and their measurement strategies (Internet). http://www.who.int/healthinfo/systems/WHO_MBHSS_2010_full_web.pdf.

[21] Ghana Statistical Service (2003). HIV PREVALENCE AND ASSOCIATED FACTORS. Ghana Demographic and Health Survey.

[22] Vonthanak Saphonn, Leng Bun Hor, Sun Penh Ly, Samrith Chhuon TS, RD (2001). How well do antenatal clinic (ANC) attendees represent the general population: A comparison of HIV prevalence from ANC sentinel surveillance sites with a population-based survey of women aged 15-49 in Cambodia. International Journal of Epidemiology.

[23] Hladik W, Masupu K, Roels T, Plipat T, Kaharuza F (2005). Prevention of mother-to-child transmission and voluntary counseling and testing programme data: what is their utility for HIV surveillance?. AIDS..

[24] Attawell Kathy (2008). Scaling up prevention of mother-to-child transmission of HIV (Internet). http://tilz.tearfund.org/webdocs/tilz/HIV/C8786_web.pdf.

[25] UNICEF (2010). Botwana Fact sheet. http://www.unicef.org/aids/files/Botswana_PMTCTFactsheet_2010.pdf.

[26] HIV/AIDS Division, Federal Ministry of Health (FMOH) (2012). HIV Program Data.

